# Lecture on Clinical Medicine, Delivered at the Philadelphia Medical Institute

**Published:** 1838-11-07

**Authors:** W. W. Gerhard

**Affiliations:** Physician to the Philadelphia Hospital, &c.


### CLINICAL LECTURE.

### LECTURE ON CLINICAL MEDICINE, *delivered at the Philadelphia Medical Institute, by* W. W. Gerhard, M. D., *Physician to the Philadelphia Hospital*, &c.

DROPSY AS A CONSEQUENCE OF DISEASE OF THE HEART.

In concluding the subject of the diseases of the heart, I have only to speak of their complications and of their terminations. You have witnessed most of these complications, and have had numerous opportunities of observing those which occur most frequently, namely, dropsical effusions. In watching the course of a heart disease, we expect that the patient will, sooner or later, become dropsical. We know that the various forms of dropsy may be called the natural termination of disease of the heart, and that when the effusion of serum is not sufficiently abundant, and does not occur in such a situation as to cause death, it is still a cause of serious inconvenience to the patient. Hence you are, as it were, obliged to be upon the alert, and must endeavour to detect this complication at its earliest appearance, and to recognise the signs by which it is preceded. That is, you must not only render yourselves familiar with the more evident phenomena of dropsy, but with those more obscure symptoms which precede the effusion of serum. Almost every important lesion is preceded by more or less constitutional disturbance before it actually manifests itself; this is the case with dropsy occurring in disease of the heart.

Besides dropsy, which may be termed the natural termination of heart diseases, these affections sometimes end prematurely, from some unforeseen accident; such, for example, as the occurrence of pneumonia, which is much more dangerous in persons labouring under a disease of the heart, than in those who were previously in good health, or what is still more sudden, death may instantly ensue from an arrest of the heart's action. This latter kind of termination is by no means rare in this class of patients; it sometimes occurs without the slightest premonitory sign; the patient may be nearly in his usual degree of health, when, from some accidental cause, the heart for a moment ceases to beat, and death immediately follows.

These accidental terminations, you have also seen: you may recollect the case of endocarditis complicated with pneumonia, which proved fatal, and you may remember how suddenly one or two patients were carried off, without having previously offered more than the usual symptoms of slight disease of the valves. My present object is not, however, to insist upon a class of sudden deaths which can rarely be foreseen, and which can almost never be prevented; nor do I propose just now to speak of the complication of heart diseases with acute inflammation of the lungs; but I will merely recapitulate the symptoms which you have recently observed in some patients who have suffered from the common, and, as it were, the regular complication of valvular disease of the heart, that is, from dropsy. I shall select for this purpose three cases, and although I have the complete observations before me, it may be more useful just now to confine ourselves to giving a condensed statement of two of them, instead of entering into details which are of great utility, but may somewhat impede our examination of the subject under the single point of view in which we are now examining it. The third case I shall give more in detail, as it occurred more recently, and is very interesting from some therapeutic difficulties which it offered.

William Elfrey, aged seventy-eight, born at Philadelphia, entered the hospital October 9, 1838. He had been a cooper for the last six years in the house, and in the habit of moderate drinking. Before admission, he was sensible of short breath *only* for three weeks; never had dyspnœa on ascending, until about that time. Cough began at the same time, with expectoration of whitish mucus. Great oppression at the epigastrium; was not conscious of palpitation, except on ascending; was unable to work for last six months, on account of the failure of his eyesight; œdema of the legs since last week; swelling of the abdomen moderate; unable to lie at night for two weeks, and obliged to sit up, inclining forwards; cough worse at night, in spells, one every hour or two; no pain, only a sensation of suffocation; has had no piles; no epistaxis or hæmorrhage from the lungs; *never ill but with ague*, seven years since, when he had had it for *five years;* never had rheumatism—occasionally a little pain in the feet. His condition at the time of his entrance was the following:—

Dark complexion; large frame, but rather emaciated; hair and teeth still preserved; yellowish, jaundiced complexion; œdema of lower extremities; position, seated; nostrils dilated; very little lividity of face; respiration forty-five, very high; pulse one hundred and eight, tolerably resisting, very irregular; the artery ossified; coolness of extremities; cough in paroxysms—has had three this morning; thin, watery, mucous expectoration; conscious of no palpitation; complained of uneasiness in epigastrium and hypochondrium, where there were evident prominence and flatness as far as the umbilicus; sounds of the heart confused—the second almost lost; impulse very moderate; first sound loud and rough; flatness below fourth rib in the præcordia complete, passing from thence to the nipple, where it became continuous with the flatness of the side; on the right side flatness complete below third rib; respiration on left side natural and expansive above fourth rib; respiration absent below upper third; posteriorly, respiration vesicular throughout left side,—feeble towards the base, where percussion was dull; on right side posteriorly, very feeble in lower two-thirds; percussion very obscure. A pediluvium was ordered, with the infusion of the melissa officinalis, and three grains of Dover's powders, and a quarter of a grain of digitalis, every three hours.

*October 10th.*—Last evening was cupped eight ounces to the præcordia; fits of dyspnœa ceased; poultice applied immediately after cupping; skin was warm after balm tea; *now* lies with head a little elevated, much less than yesterday; less oppression; respiration twenty-three, less elevated, by an alternate movement of the ribs and diaphragm; pulse ninety-two, fuller, less irregular; skin cool at extremities, but less so than before; face a little livid, with permanent dilatation of the nostrils; tongue a little blue, moist, clean; still dizziness; intellect less dull; urine evidently increased—two or three discharges this morning; respiration, left side, anteriorly, louder and fuller; on the right side, much louder—feebleness of it extending scarcely above the nipple; dulness on percussion to the same extent; impulse of heart very moderate; both sounds heard, something like the double tick of a watch; creaking of parchment doubtful yesterday, clear to-day; percussion flat to fourth rib; posteriorly, respiration, right side, inferiorly, rude; percussion much clearer, a little dull at base. Balm tea, Dover's powders, and digitalis, continued; poultice.

*October 12th.*—Pulse one hundred, less irregular; five paroxysms from twelve o'clock, yesterday, until the evening; skin more natural; no sweating; can lie with his head at an angle of forty degrees; œdema of the legs much less; no swelling of the abdomen; cough less severe; no soreness at epigastrium, less tension; lips less injected; bronzed colour of the skin less marked; tongue moist, still somewhat purplish; respiration on right side anteriorly, vesicular and feeble, at lower fourth, almost absent—throughout, a little harsh; left side louder than on right, vesicular—absent over præcordial region; creaking now very loud at the level of the valves, evidently not valvular sounds; can be felt, as well as heard; extends to the sternum, a space one and a half inches square; sounds of the heart louder; second almost lost, accompanied by bruit de cire; rhythm altered; impulse stronger than average; to the right of the sternum both sounds heard a little altered; percussion dull over the fourth rib, to an inch beyond the nipple, where it is lost in the axilla, perfect near the sternum, extending to right margin; respiration posteriorly left side, at the inferior one-third absent; upper two-thirds vesicular, nearly natural; right side, absence of respiration almost complete in lower one-half, slightly bronchial; œgophony on both sides corresponding to the level of the liquid; higher on right than left; continue treatment.

13*th.* Slept much better than before; had two or three paroxysms last night, none this morning. Lies with his head much lower; pulse eighty-four, very irregular; thinks a paroxysm is coming on; asks for powder, thinks they shorten the paroxysm; dyspnœa much less. Some rasping in the first sound of the heart; absence of second almost complete at the semilunar valves; creaking occasionally heard, most loud over the middle of the heart, both in the systole and diastole. During auscultation, a paroxysm coming on; action of heart becomes quicker, spasmodic; the bruit de cire more constant, louder; impulse increased; same sound heard in both sounds, but less loud in second; powders continued every four hours.

15*th.* Sleep has been very good since last date; can lie on either side, with his head very low; oppression almost gone; pulse sixty-eight, less irregular; no cephalalgia; no pain any where; cough very slight; tongue moist, scarcely coated; urine abundant, about three quarts daily; no sweating; abdomen flaccid; tension at hypochondrium almost gone; no swelling of the feet; tongue moist, but still of a purple tint, as well as the lips; top of skin generally less bronzed; two stools; respiration easy, fourteen; respiration on left side anteriorly vesicular, almost natural, except at the base of the axilla, heard distinctly over the heart; impulse of the heart much stronger; strong rasping sound in the first; second almost lost; a little creaking heard at times. Percussion sonorous below fourth rib; flatness from thence continuous with the region of the pleura, not connected with the pericardium; posteriorly, respiration vesicular throughout; powders continued morning and night; infusion of juniper berries; full diet.

17*th.* Decubitus low, nearly horizontal; pulse sixty, irregular; respiration twenty; tongue less livid; lips less so; urine continues copious—between three and four quarts—no swelling; cephalalgia better; two stools daily; powder once daily; continue infusions.

22*d.* Convalescence has continued; the patient is walking about; full diet given after the first four days; walks up stairs without difficulty; very little shortness of breath; no cough; no pain.

*Auscultation* of the heart strongly rasping, severe in the first sound over verge of the semilunar valves; a little grating heard towards the apex; and different from the action of the valves; on right side of sternum, where the second sound is most developed, the rasping continues; impulse at the top of the sternum strong; a single rough sound, which is synchronous with that heard at valves; and second transmitted along aorta; impulse evidently distinct; respiration dull at upper third of sternum; flat at lower two-thirds extending to nipple, but not to axilla; not to right of sternum.

Discharged, cured of the acute affection. There remains chronic disease of the valves of the aorta with hypertrophy and dilatation, with patches of lymph in the pericardium.

This patient was employed, as you perceive, in the out-wards of the institution, and suffered so little inconvenience from his disease of the heart, as to be quite unconscious of its existence; there were neither dyspnœa nor palpitations sufficiently severe to prevent him from following his ordinary occupation. He is, however, afterwards attacked with a new complication: that is, acute inflammation of the pericardium, and in a slight degree of the endocardium. This complication at once increases the dyspnœa, offers an additional impediment to the circulation of the blood through the lungs and heart, and is quickly followed by dropsical effusions.

When the patient entered the hospital there was œdema of the limbs, and effusion into both the pleuræ and the pericardium, that is hydrothorax; we were able to trace the quantity of liquid then contained in the cavities, and we could also estimate the rapidity of its absorption, by the gradual subsidence of the line of dullness. By physical examination we could go still further in our diagnosis and ascertain that the heart was at first separated to some distance from the walls of the thorax, by the liquid effused into the pericardium, and that in proportion as the effusion diminished, we heard a grating sound at the præcordial region. This creaking sound was a proof that the effusion into the pericardium was not simply dropsical, but that it depended in part, at least, upon an inflammatory action in this cavity.

You have observed how intense the dyspnœa appeared at the entrance of the patient; he was obliged to sit up and lean forward, breathing with extreme effort, and obviously labouring under an almost complete stagnation of the circulation. The heart performed its functions with great difficulty, its cavities were surcharged, and the lungs were therefore over-loaded. The same impediment to the circulation, gave rise to coldness, lividity, and œdema of the extremities. The patient was obviously in danger; the circulation required to be relieved, and at the same time, in the effort to diminish the quantity of blood in the heart, we were bound to avoid increasing the feebleness of the patient, for in these cases a very slight abstraction of blood may do harm. I therefore directed sinapisms to be placed upon the extremities, with a warm infusion of the common balm, as a drink. Dover's powders, combined with digitalis, were to be given internally, but these remedies could not, of course, produce any immediate effect. I directed cups to be applied, if the circulation should again become more vigorous; the sinapisms and warm drinks were followed by some relief; but the patient was not cupped until complete reaction had occurred; he was immediately relieved by the cupping, and continued to improve, until the effusion had completely disappeared.

When you meet with cases of this kind, you may generally relieve them, provided they have occurred suddenly; if, however, the dropsy has come on more slowly, and is connected with an enfeebled state of the system and a diminution of the quantity of red globules in the blood, you will meet with much more difficulty. When the patient has not completely lost the vigour of his constitution, he may readily react from the temporary depression caused by the impediment to the circulation, but it is always expedient first to resort to measures designed to excite the circulation in the exterior of the body, such as fomentations, sinapisms, stimulating pediluvia, and warm drinks. You may afterwards, as soon as the circulation has become more equalized, unload the heart and large vessels, by taking blood from the arm, by free cupping over the præcordial region, or between the shoulders. When you have succeeded in your treatment, and have diminished the dyspnœa, you may place the patient upon the use of digitalis. As you may have remarked, I frequently combine this remedy with Dover's powders, and in this way tranquillize the action of the heart more effectually than could be done by the digitalis alone.

One of the other cases to which I have alluded, is now in the wards. It is Wise, a patient who labours under the unfortunate complication of phthisis, and dilatation and hypertrophy of the heart, with its attendant disease, general dropsy. The tuberculous disease is chiefly confined to the right lung, which is adherent throughout a considerable part of its extent. Now, the effused serum cannot accumulate in the right pleura, in consequence of these adhesions, and it is therefore collected in such quantities on the left side as to give rise to the most distressing dyspnœa. It is also secreted in considerable quantities in the pericardium. Of course, the disease of the right lung diminishes the quantity of pulmonary tissue still permeable to the air; the patient, therefore, suffers doubly from the hydrothorax.

The dropsy extends to the lower extremities, as well as the parts of generation, and annoys the patient very much from the pain which he suffers in the scrotum and penis. The anasarca commenced suddenly after making a violent effort in lifting a heavy beam. From the symptoms offered by the patient after his admission, there was reason to believe that in this case, as well as in that just related, the dropsy coincided with an acute inflammation of the heart, occurring during the course of chronic hypertrophy. The patient is still in great danger, and the treatment is necessarily difficult from the numerous complications of the disease, which require to proceed with extreme caution. I am now carefully puncturing the thighs and scrotum, applying bandages to the legs, and giving the patient a pill three times daily, composed of three-fourths of a grain of digitalis, a sixteenth of a grain of calomel, and a third of a grain of opium. He takes also an infusion of juniper berries, as a ptisan.

The third patient is a mulatto, named Henry, who was twice under treatment. On the first occasion, anasarca came on simultaneously with pericarditis. He was entirely cured of the dropsy, and the disease of the heart was reduced to simple hypertrophy and dilatation. The man left the hospital, commenced a laborious employment of the most unsuitable kind, drawing a hand-cart, and was exposed to a shower of rain. The next day after this wetting, he was attacked with dyspnœa and palpitation, and in a day or two afterwards was obliged to enter the ward for a second time; the patient was again treated for anasarca, with little success; ascites afterwards supervened, he was tapped, suffered little inconvenience from the operation, and, after a partial improvement, was carried off suddenly, without any previous increase in the alarming symptoms. Upon examination, the heart was found to be enlarged, it was thickened, and its cavities were dilated to nearly twice their natural size; the valves were nearly in the natural state. Organized patches of lymph and partial adhesions, were found in the pericardium. I mention this case, that you may again remark, how frequently acute inflammation of the membranes of the heart complicates a chronic disease, and proves the exciting cause of dropsical effusions.

NOTE.—These lectures on diseases of the heart, form part of a series which were given upon this subject. The remainder cannot be published at present, from the necessity of following the regular winter lectures, of which reports will be given in the Examiner. Of the whole course delivered during the summer, about one-third has been published; it is intended to complete it as soon as practicable. The lectures published previously to the month of September, were printed from notes taken at the time they were delivered; these notes were, with the exception of two or three lectures, revised by the lecturer. The latter part of the course, consists of sketches furnished by the author. This explanation seems necessary, to account for the peculiarities which may be perceived in the phraseology of different parts of the course.

---

*Lithotomy.*—On the 13th ult., Professor Dudley, of Lexington, Kentucky, performed his 157th operation of Lithotomy. The patient was a boy of ten years of age, and was the thirteenth case from the same county.

---

M. Fricke, of Hamburgh, recommends nitrate of silver to be rubbed over frost-bites.

